# Gene Expression Data for Investigating Glaucoma Treatment Options and Pharmacology in the Anterior Segment, State-of-the-Art and Future Directions

**DOI:** 10.3389/fnins.2022.912043

**Published:** 2022-06-08

**Authors:** Georg Fuellen, Anselm Jünemann

**Affiliations:** ^1^Institute for Biostatistics and Informatics in Medicine and Aging Research, Rostock University Medical Center, Rostock, Germany; ^2^Department of Ophthalmology, Rostock University Medical Center, Rostock, Germany; ^3^Department of General Ophthalmology and Pediatric Ophthalmology Service, Medical University of Lublin, Lublin, Poland

**Keywords:** glaucoma, anterior segment, omics, gene expression, trabecular meshwork, tenon

## Abstract

Glaucoma treatment options as well as its etiology are far from understood. Gene expression (transcriptomics) data of the anterior segment of the eye can help by elucidating the molecular-mechanistic underpinnings, and we present an up-to-date description and discussion of what gene expression data are publicly available, and for which purposes these can be used. We feature the few resources covering all segments of the eye, and we then specifically focus on the anterior segment, and provide an extensive list of the Gene Expression Omnibus data that may be useful. We also feature single-cell data of relevance, particularly three datasets from tissues of relevance to aqueous humor outflow. We describe how the data have been used by researchers, by following up resource citations and data re-analyses. We discuss datasets and analyses pertaining to fibrosis following glaucoma surgery, and to glaucoma resulting from the use of steroids. We conclude by pointing out the current lack and underutilization of ocular gene expression data, and how the state of the art is expected to improve in the future.

## Significance Statement

The use of omics data in ophthalmology needs to be fostered, so that more detailed molecular-mechanistic insight becomes available in the future. Our minireview provides some important support in this direction by describing what gene expression data are available and how useful these are, focusing on glaucoma research in the anterior segment of the eye. Our minireview demonstrated that the ocular gene expression data landscape is growing, as more datasets, in particular with single-cell resolution, as well as some more multi-tissue resources are being published. While previous datasets were often rarely utilized, for the newer datasets higher utilization rates are expected, as the increases in coverage and detail, as well as new analysis methods, render omics data ever more valuable for improving our molecular-mechanistic understanding of glaucoma. While gene expression data regarding the posterior segment of the eye were reviewed by others a year ago, we fill the gap for the anterior segment.

## Introduction

Glaucoma, cataract, age-related macular degeneration and diabetic retinopathy are the main causes of visual impairment and, in the worst case, blindness. These conditions decrease or even destroy quality-of-life and are associated with huge medical, societal, and economic costs. Glaucoma is a neurodegenerative disease that is, however, often caused by filtration failure of the trabecular meshwork and outflow system, leading to high intraocular pressure by excess aqueous humor. Glaucoma treatment usually entails the lowering of intraocular pressure by eye-drops or by surgery designed to improve existing filtration pathways, or to create alternative outflow pathways for the aqueous humor. Yet, treatment options are limited. Eye drops often fail in the long run, and improvements in aqueous humor outflow are often diminished by fibrotic processes (Georgoulas et al., 2008; Schlunck et al., 2016). Further downstream, glaucoma treatment by tackling the neurodegeneration affecting the retina, in particular the retinal ganglion cells, due to high intraocular pressure is a promising field of future research. Along these lines, gene expression analyses of glaucomatous neurodegeneration were reviewed very recently (Wang et al., 2021) and also a few years back (Nickells and Pelzel, 2015). Nevertheless, we note that as the most comprehensive accomplishment at the time of writing, a multi-omics single-cell dataset of the human retina has recently become available as a preprint (Liang et al., 2021). Moreover, regarding the cornea, the first large-scale single-cell dataset was just published (Collin et al., 2021). Finally, a recent meta-analysis of glaucoma-related gene expression yielded drug candidates protecting retinal ganglion cells, as described by Enz et al. (2021).

More knowledge of the molecular processes behind glaucoma treatment options is thus needed. One approach to add knowledge is the generation and analysis of high-throughput (omics) data, that is, measuring methylation marks, transcripts, proteins, metabolites or other molecular entities in a rather comprehensive, large-scale manner. These data may then give rise to disease- or treatment-related biological processes or pathways. This mini-review is intended to summarize and discuss the gene expression (transcriptome) data currently available for molecular investigations in ophthalmology, with a focus on the anterior segment of the eye, and a specific focus on the trabecular meshwork and conjunctival Tenon’s capsule, which are the areas where the progression from the healthy to the glaucoma state usually starts, and where the fibrosis of the conjunctival filtration surgery area is originating, respectively. As described, a review with a corresponding focus on the posterior segment of the eye was published in 2021 (Wang et al., 2021).

## Gene Expression Data Enable Molecular Insights

Many times, high-throughput (omics) data were found to be an invaluable tool in gaining molecular-mechanistic insights, despite their inherent noisiness due to biological variability and measurement challenges (Tanoli et al., 2021). Here we focus on measuring gene expression (that is, transcriptomics). Microarray technology is now in use for more than two decades, and Next-Generation-Sequencing (NGS, or RNA-seq) for around one decade; the former is limited to defined probesets of genes, so that only the latter enables an unbiased and often more accurate view of anything that is transcribed in cells or tissues. In particular, gene expression data hold promises to enable the identification of molecular biomarkers, which may be diagnostic, prognostic, or predictive of treatment outcome. Genetic data are usually the most accessible in humans, while other omics such a gene or protein expression are closer to physiology (and they are dynamic); quantitative trait locus data (QTLs, e.g., from GTEx) can mediate between these two (Sieberts and Schadt, 2019). Due to their noisiness, omics data are usually aggregated to obtain robust insights. The most over- or underexpressed genes (out of the roughly 20,000 genes/transcripts usually available) are often “novel findings” for the disease or condition under investigation, and they may well be subject to fluctuations. However, aggregating genes into gene sets or pathways and checking their enrichment among the over- or underexpressed genes frequently matches existing knowledge and/or can be interpreted in a meaningful way (Tanoli et al., 2021).

## Gene Expression Data of the Eye, With a Focus on the Anterior Segment

### Ocular Transcriptomics Data Covering Multiple Tissues Incl. the Posterior Segment

There is a lack of ocular omics data, because tissues or cells from the eye are not usually covered by the major collaborative omics-related projects (Paananen and Fortino, 2020). In a recent review, Owen and Moosajee (2019) tabulated ocular RNA-Seq datasets only for a few conditions or tissues, and these refer almost exclusively to the cornea and the retina. Furthermore, it is often unclear in how far these data, established *in vitro* or from biopsies or from model organisms, truly reflect the *in vivo* situation in humans. In case of interventions, human data are sometimes available, limited, however, to the few data becoming available as part of clinical trials.

For some time, the one major large-scale dataset available for the eye consisted of microarray data of 10 human eye tissues, that is, retina, optic nerve head, optic nerve, ciliary body, trabecular meshwork, sclera, lens, cornea, choroid/retinal pigment epithelium (RPE), and iris. Made available by the OTDB database (GSE41102)^1^ (Wagner et al., 2013) straightforward “Is-gene-X-expressed-in-tissue-Y?” type of queries are enabled using its web interface. More recently, the *Eye in a Disk: eyeIntegration* (Swamy and McGaughey, 2019) resource featured human cornea, eyelid, lens, retina, retinal epithelium and RPE (choroid). By the same group, but exclusively focusing on retinal tissues, the *Mega Single Cell Transcriptome Ocular Meta-Atlas* (Swamy et al., 2021; BioRxiv) was created. Finally, a recently developed database is the eye-transcriptome.com dataset (Wolf et al., 2022), consisting of 10 healthy human (conjunctiva, cornea, eyelid, lacrimal gland, optic nerve, retina periphery, retina center, choroid/RPE, retinal microglia, and hyalocytes from the vitreous) and 9 diseased human tissues, where all data stem from the authors’ institution. Among the eye tissues covered by the major resources listed here, the trabecular meshwork and the conjunctiva have the highest relevance to glaucoma research in the anterior segment of the eye. The tissue coverage of the large-scale resources is featured in Table 1, together with the tissue coverage of the single-cell and GEO resources mentioned in the following paragraphs.

**TABLE 1:** **(A)** Overlap of tissues described in ocular gene expression datasets.

	Retina	Choroid/retinal pigment epithelium		Optic nerve head		Optic nerve	Ciliary body		Trabecular meshwork		Sclera
*OTDB*	*x*	*x*		*x*		*x*	*x*		*x*		*x*
*Eye integration*	*x*	*x*									
*eye-transcriptome.com*	x***	*x*				*x*					
Single-cell*							*x*		*x*		
GEO**									*x*		

	**Lens**	**Cornea**	**Iris**	**Eyelid**	**Conjunctiva/tenon**			**Lacrimal gland**		**Vitreous (hyalocytes)**	

*OTDB*	*x*	*x*	*x*								
*Eye integration*	*x*	*x*		*x*							
*eye-transcriptome.com*		*x*		*x*	*x*			*x*		*x*	
Single-cell*					*x*						
GEO**					*x*						

**TABLE 1** | **(B)** Source species of ocular gene expression datasets.				

	**Human**		**Mouse**		**Other**		**Web interface**					

*OTDB*	*x*						*genome.uiowa.edu/otdb/*					
*Eye integration*	*x*						*eyeintegration.nei.nih.gov*					
*eye-transcriptome.com*	*x*						*eye-transcriptome.com*					
Single-cell*	*x*		*x*		Monkey/pig		*(in part) www.ncbi.nlm.nih.gov/geo/*					
GEO**	*x*		*x*		Rabbit		*www.ncbi.nlm.nih.gov/geo/*					

*As featured in this text, anterior segment only. ** As featured in this text, anterior segment only (see also Supplementary Text 2). ***Retina periphery, retina center, retinal microglia.

Overall, at the time of writing (December 27, 2021), the OTDB (Wagner et al., 2013) was cited 102 times, and to find out about its use in ocular treatment investigations, we did (fulltext) google scholar searches within the papers citing the OTDB paper, using the query *pharmacology OR “drug development” OR pharmacokinetics OR pharmacodynamics*, which yielded 18 papers. Tracking down the citations and going through the papers or dissertations for which full-text was readily available, we found 6 uses of OTDB for confirmation of an expression pattern, two citations without explicit use of the database, and one comparison of OTDB to a resource of retinal data. As far as we could see, the other (much more recent) resources of the last paragraph have not yet been followed up in such ways.

Further demonstrating the “underutilization” of ocular omics data, the review of Owen and Moosajee (2019) consists of some introductory examples of the use of ocular omics data that were generated with a specific question in mind, technical notes, a table of existing resources, a visualization example, and concluding sections on the “Utility of RNA-seq in ophthalmology research” that are, however, mostly descriptive; in one exceptional case, similarity on the omics level suggested the validity of a model system, and in the other exceptional case, omics data were used to validate a research hypothesis. Then again, the increasing adoption of FAIR data principles (Wilkinson et al., 2016; Wise et al., 2019) and the increasing precision, sophistication, and utility of the omics data types (such as single-cell data, see below) and methods of analysis and integration (such as deep learning or transfer learning) (Kowald et al., 2022) for an increasing number of ocular tissues for more and more species, give hope that ophthalmology will benefit from omics data efforts similarly to fields such as cellular senescence (Zhu et al., 2015).

### Ocular Transcriptomics Data of the Anterior Segment of the Eye

With a focus on glaucoma research in the anterior segment of the eye, we were most interested in the anterior chamber, and particularly the trabecular meshwork (through which a major fraction of the aqueous humor exits the eye), the tenon tissue (Tenon’s capsule, hosting the fibroblasts that trigger the scarring after glaucoma surgery), and the conjunctiva as well as the ciliary body, which are closely neighboring tissues. We were also interested in primary cell cultures or cell lines derived from these. Therefore, we explored the GEO database, searching for data from the anterior chamber (MeSH Term), ciliary body (MeSH Term), conjunctiva (MeSH Term), trabecular meshwork (MeSH Term), and tenon (All Fields). The complete search term (Supplementary Text 1) was further designed to ignore datasets featuring exclusively measurements of methylation or non-coding RNA. On December 27, 2021, we obtained 289 records, from which we kept 61, manually filtering out spurious hits that exclusively featured, e.g., cornea, iris, lens or retina/nervous tissue. We also ignored embryonal data, as well as cancer-only and *Xenopus* data. Mentionings in the following are sorted by tissue and research topic, and otherwise in reverse chronological order. Unless the data are single-cell data, we only report datasets where at least for one condition, replicates were done.

#### Single-Cell Data of the Anterior Segment

Single-cell data may carry the most detailed information, and some newer GEO datasets and corresponding papers report single-cell data of interest. Three of these, that is, GSE168200 (Thomson et al., 2021) for mouse, GSE146188 (van Zyl et al., 2020) for human/monkey/mouse/pig, and PRJNA616025 (no GSE identifier) (Patel et al., 2020) for human consider aqueous humor outflow pathways (focusing on the trabecular meshwork and Schlemm’s canal), while others consider ciliary epithelium including fibroblasts (GSE178667; Youkilis and Bassnett, 2021, for mouse), and myeloid cells of, among other eye tissues, the conjunctiva (GSE160797; Wieghofer et al., 2021, also for mouse). While single-cell data can give a much more detailed description of a tissue, their coverage of eye tissues is still limited, use of such data is still in its infancy, and their long-term translational value still needs to be demonstrated.

#### Bulk Gene Expression Data of the Anterior Segment, General Remarks

In the following, we describe bulk gene expression data obtained from the tissues of interest. Although some of these data are around for a long time, their translational value is as underutilized as already noted for the OTDB; most utilized are some datasets of the trabecular meshwork that were specifically generated to explore its degeneration caused by the use of steroids, to *explain* glaucoma as a side effect of steroid use. However, as we will see, these data were essentially all used to generate hypotheses about the molecular mechanisms behind differences in the effect of some drugs under various conditions; their predictive use is currently lacking, to the best of our knowledge.

#### Bulk Gene Expression Data, Trabecular Meshwork

The *trabecular meshwork (TM)* was the subject of a remarkable number of studies, by microarray and more recently by RNA-seq. In GSE180407 (Filla et al., 2021), the effects of overexpressing either a wild type or a constitutively active avb3-integrin on the transcriptome of an immortalized line of human TM cells are described. GSE159793/GSE122652 (Nettesheim et al., 2019; Shim et al., 2021) investigate human TM cells with and without silenced expression of the autophagy genes Atg5 and Atg7, some of which were subjected to cyclic mechanical stretch. In GSE123100 (Tie et al., 2020), primary human TM cells are cultured on polyacrylamide gels with tunable stiffness and their gene expression is compared with gene expression in glaucomatous TM tissues. GSE138125, GSE27276 (Liu et al., 2013) and GSE27058/GSE27057/GSE4316 consider the simplest experimental setup, of human TM from control vs. glaucoma patients, but a matching set of lncRNA data is also provided by GSE27276. GSE124114 (Faralli et al., 2019) features data from dexamethasone-treated and control human TM cells. Similarly, GSE65240 (Matsuda et al., 2015) and GSE37474 are about dexamethasone (or vehicle) treatment of human TM (cells or tissue/organ culture), while GSE16643 (Nehme et al., 2009) reports on dexamethasone, fluocinolone acetonide and triamcinolone acetonide. Further human TM cellular intervention studies are reported by GSE40314 (FOXC1 knockdown), GSE32169 (Porter et al., 2012) (phagocytic challenge under physiological and oxidative stress conditions), GSE27275 (PITX2 knockdown), GSE18713 (Luna et al., 2009b) (miR-29b), GSE14768 (Luna et al., 2009a) (cyclic mechanical stress), GSE6298 (Fan et al., 2008) (dexamethasone and triamcinolone), and by GSE7144 (TGF-beta1 and 2). While the studies on the effects of dexamethasone and other steroids on the TM are currently among the best examples of omics data use in ocular pharmacology, they were only exploited in an explorative fashion by now, providing hypotheses for mechanisms of action under a variety of conditions (see also below).

#### Bulk Gene Expression Data, Trabecular Meshwork Measured Together With Related Tissues

Enabling the comparison between tissues/cells, human TM, cornea, sclera and TM-derived mesenchymal stem cells were investigated in GSE87526 (Sathiyanathan et al., 2017); human TM and other eye tissues, pooled from several individuals, are also part of the OTDB dataset already mentioned above (GSE41102; Wagner et al., 2013). Similarly, data of human sclera fibroblasts, choroidea fibroblasts, and Tenon’s space fibroblasts were generated and analyzed, see GSE40929 (Lobler et al., 2013). Further, gene expression of TM-derived cells was measured together with gene expression of other somatic cell types, including scleral and corneal fibroblasts, and reported in GSE28679. Finally, human ciliary muscle, and human TM cells were treated with the prostaglandin analogs *latanoprost free acid* or *prostaglandin F2alpha*, see GSE492. In Supplementary Text 2, further multi-tissue GEO datasets of the anterior segment that are not featuring the TM, and in Supplementary Text 3, further GEO datasets from the conjunctiva, but not featuring the tenon are described.

#### Bulk Gene Expression Data, Conjunctiva/Tenon

The datasets reported up to now may support analyses of the TM, which may degenerate and become fibrotic, giving rise to glaucoma. As described, another case of glaucoma-related fibrosis happens after glaucoma surgery (e.g., trabeculectomy), and here, tenon fibroblasts are most often implicated; these are considered to be part of the conjunctiva. In this context, RNA-seq gene expression data (for rabbit) in a time series after trabeculectomy are reported by GSE156781 (Fujimoto et al., 2021); RNA was isolated from rabbit *conjunctiva*, including filtering blebs. Of note, not found in GEO is a matching mouse dataset (Adachi et al., 2020), where the glaucoma filtration surgery model consisted of an incision of the limbal conjunctiva, followed by the insertion of a 33G needle tip into the anterior chamber, and 11–0 nylon sutures; RNA was taken from bleb region tissue and compared to control conjunctiva. Unfortunately, the supplementary tables for their paper only feature a selected subset of the processed data. Finally, for human, a milestone for glaucoma omics are the RNA-seq data by Yu-Wai-Man et al. (2017), which are the single glaucoma dataset featured by the review cited above (Owen and Moosajee, 2019). These data are based on fibroblast cell lines from explants from glaucoma surgery done first-time, or redone after failure. Expression values of a subset of 246 genes were made publicly available together with the paper; unfortunately, there is no public raw data. The 246 genes were subjected to gene ontology enrichment analyses and assembled into a network based on public gene/protein interaction and pathway data, and subnetworks were inspected toward a better mechanistic understanding of the molecular processes behind fibrosis as they may contribute to the failure of glaucoma surgery.

#### Bulk Gene Expression Data, (Re-)analyses of Public GEO Datasets on Steroid Response

(Re-) analyses of public Gene Expression Omnibus (GEO) datasets are published at times; for example, differential gene expression induced by dexamethasone in the anterior segment of the human eye (GSE37474) was reinvestigated (Zhang et al., 2019), featuring gene ontology enrichment and gene/protein interaction network-based results. A more comprehensive re-analysis was done by integrating the GSE37474 data with the datasets GSE6298 (Fan et al., 2008), GSE16643 (Nehme et al., 2009), GSE65240 (Matsuda et al., 2015), and GSE124114 (Faralli et al., 2019), and reported by Liesenborghs et al. (2020). These authors also added a bovine RNA-sequencing dataset in which a distinction between eyes with and without phenotypically estimated corticosteroid response was made (Bermudez et al., 2017). A similar human dataset is also described in Bermudez et al. (2017). In an integrative analysis, significantly changed pathways and functional categories were considered, highlighting collagen, extracellular matrix, adhesion, WNT-signaling, inflammation, adipogenesis, and glucose metabolism. In addition, cell cycle and senescence were found to be significantly changed in corticosteroid responders vs. non-responders, based on the bovine data.

The two re-analyses just described both deal with glaucoma due to steroids, and they are, joint with the original datasets, among the best examples of the use of any of these omics data in ocular pharmacology and drug development, at least in an explorative, descriptive fashion. Two reviews published some years ago confirm this. Based on a review of pharmacokinetic and gene activation profile data of glucocorticoids in ophthalmology, one review (Whitcup et al., 2018) concludes that understanding basic science shall make it possible to select the best therapeutic agent for patients and to discover novel corticosteroids with even better safety and efficacy profiles for ophthalmic disease. The other review (Fini et al., 2017) focusses on pharmacogenomics (including genome-wide association studies) with functional follow-up, toward identifying new drug targets for ocular hypertension and providing a predictive and diagnostic tool for use of drugs; here, gene expression, pathway, and network data are deemed valuable for their ability to pinpoint detailed mechanisms. In our repositioning of an antibiotic as an antifibrotic compound after glaucoma surgery (Stahnke et al., 2020), we described one of the few predictive uses of gene expression data in ocular drug development; *in vivo* validation is still lacking, however. (We deposited our gene expression data at the European Nucleotide Archive, PRJEB38998, not in GEO, as per the recommendations of the journal.)

## Discussion

Overall, tallying the existing data demonstrated that apart from the 2013 OTDB resource, until about 2021 it was mostly single GEO datasets that dominated the ocular transcriptomics landscape, and these data were rarely utilized. More recently, however, several comprehensive resources popped up, including *eyeIntegration* (Swamy and McGaughey, 2019) and eye-transcriptome.com (Wolf et al., 2022) along with more specialized single-cell datasets (Patel et al., 2020; van Zyl et al., 2020; Thomson et al., 2021; Wieghofer et al., 2021; Youkilis and Bassnett, 2021). For the future, several groups are working on more comprehensive single-cell datasets of the eye, and on other omics modalities, so that a surge of future data generation and utilization can be expected, and as long as data are deposited under FAIR criteria whenever possible, a bright future is in sight.

## Author Contributions

GF wrote the manuscript. AJ critically revised the manuscript. Both authors contributed to the article and approved the submitted version.

## Conflict of Interest

The authors declare that the research was conducted in the absence of any commercial or financial relationships that could be construed as a potential conflict of interest.

## Publisher’s Note

All claims expressed in this article are solely those of the authors and do not necessarily represent those of their affiliated organizations, or those of the publisher, the editors and the reviewers. Any product that may be evaluated in this article, or claim that may be made by its manufacturer, is not guaranteed or endorsed by the publisher.

## Footnotes

1
genome.uiowa.edu/otdb/
