# Supplementary material for: Gene Expression Data for Investigating Glaucoma Treatment Options and Pharmacology in the Anterior Segment, State-of-the-Art and Future Directions
Source: Front Neurosci. 2022 Jun 8;16:912043. doi: 10.3389/fnins.2022.912043 (PMC9213806; doi:10.3389/fnins.2022.912043)
Supplement: Supplementary file 1 [file Data_Sheet_1.docx]

***Supplemental Texts:***

**Minireview: Gene expression data for investigating glaucoma treatment options and pharmacology in the anterior segment, state-of-the-art and future directions**

***Supplemental Text 1.*** To retrieve the GEO DataSets, the following query was constructed using the GEO *Advanced Search Builder.*

(("Anterior Chamber"[MeSH Terms] OR "Ciliary Body"[MeSH Terms] OR ("conjunctiva"[MeSH Terms] OR Conjunctiva[MeSH Terms]) OR "Trabecular Meshwork"[MeSH Terms] OR tenon[All Fields]) AND (("expression profiling by array"[DataSet Type] OR "expression profiling by genome tiling array"[DataSet Type] OR "expression profiling by high throughput sequencing"[DataSet Type] OR "expression profiling by mpss"[DataSet Type] OR "expression profiling by rt pcr"[DataSet Type] OR "expression profiling by sage"[DataSet Type] OR "expression profiling by snp array"[DataSet Type]) OR ("genome variation profiling by array"[DataSet Type] OR "genome variation profiling by genome tiling array"[DataSet Type] OR "genome variation profiling by high throughput sequencing"[DataSet Type] OR "genome variation profiling by snp array"[DataSet Type])) OR ("other"[DataSet Type] OR "protein profiling by mass spec"[DataSet Type] OR "protein profiling by protein array"[DataSet Type] OR "snp genotyping by snp array"[DataSet Type] OR "third party reanalysis"[DataSet Type])))

***Supplemental Text 2.*** Further GEO datasets of interest: Multi-tissue of the anterior segment but not featuring the trabecular meshwork.

Multi-tissue datasets of the anterior segment but not featuring the trabecular meshwork are GSE38190 (Nakatsu et al., 2013), which refers to gene expression data from human conjunctiva, limbus and cornea, GSE26076 (Ramirez-Miranda, Nakatsu, Zarei-Ghanavati, Nguyen, & Deng, 2011), which reports human conjunctiva and cornea gene expression, GSE12604 (Majo, Rochat, Nicolas, Jaoude, & Barrandon, 2008), which reports pig conjunctiva and cornea keratinocyte gene expression, GSE8633 (Tong et al., 2009), which features human conjunctival tissue as well as primary conjunctival epithelial cells, IOBA-NHC and ChWK conjunctival epithelial cell lines, and GSE5543 (Turner, Budak, Akinci, & Wolosin, 2007), which features human conjunctival and corneal epithelia. Pigmented and non-pigmented epithelia of the *ciliary body* of human healthy eyes were sequenced and made available via GSE37957 (Janssen et al., 2012). Further, ciliary body, cornea, lens, iris, retina, and optic nerve underwent gene expression analysis as reported in GSE3023 (Diehn, Diehn, Marmor, & Brown, 2005). Whole-eye bulk data are rare; GSE106591 (Barbato et al., 2017) provides whole-eye (after removal of lens and cornea) data, in miR-211^-/-^ mice and controls.

***Supplemental Text 3.*** Further GEO datasets of interest: Conjunctival datasets not featuring the tenon.

Conjunctival datasets not featuring the tenon can also be found in GEO. Referring to human ***conjunctiva***, GSE149004 (Boneva et al., 2020) compares standard RNA-seq (from fresh tissue) to 3’ massive analysis of c-DNA ends (MACE) RNA-seq (from fresh and FFPE-conserved tissue), reporting few degeneration problems in case of FFPE. GSE148387 (Martin et al., 2021) features MACE RNA-seq data by the same research group, for another set of human conjunctiva samples. Also available are human *conjunctiva* of Sjogren Syndrome keratoconjunctivitis sicca and controls (GSE176510, Nanostring data), human conjunctiva of congenital aniridia patients and controls (GSE137997, mRNA and miRNA data (Latta et al., 2021)), rabbit conjunctiva of a dry eye disease model (GSE171043 (Master et al., 2021)), conjunctiva from Ethiopians after trichiasis surgery (GSE135455 (Derrick et al., 2019); good and poor outcome are distinguished for doxycycline versus placebo treatment), conjunctiva from Solomon Islanders with trachoma (GSE106961; disease/infection versus controls), as well as from Tanzanians (GSE24383; disease/inflammation versus controls), Ethiopians (GSE23705; disease/inflammation versus controls) and Gambian children (GSE20436/GSE20430; disease versus controls) (Roberts et al., 2015), (Hu et al., 2012).

Further, GSE111496 (Chen et al., 2018) makes available data from immortalized human conjunctival (among others) cells pre-treated with dihydrotestosterone or vehicle, then exposed to lipopolysaccharide and ligand binding protein or vehicle; GSE83627, GSE51995 (Hou et al., 2014) and GSE2513 (Wong, Chew, Yang, Tan, & Beuerman, 2006) feature pterygium and conjunctiva tissues obtained from four patients each; GSE77361 features conjunctiva whole tissue and primary conjunctival fibroblasts from patients with ocular mucous membrane pemphigoid and control patients, GSE28941 describes conjunctival epithelium of patients with severe dry eye disease and controls; GSE18094 (Khandelwal, Liu, & Sullivan, 2012) employs immortalized human conjunctival epithelial cells subjected to dihydrotestosterone; and, finally, GSE2835 (Hori, Spurr-Michaud, Russo, Argueso, & Gipson, 2005) employs a human conjunctival epithelial cell line treated with retinoic acid. Further, goblet cells of the mouse *conjunctiva* were investigated in Spdef^-/-^ and control mice, see GSE44101 (Gipson, 2016); conjunctival forniceal epithelial cells in Klf4-conditional null and control mice, see GSE26076 (Gupta, Harvey, Kaminski, & Swamynathan, 2011); and a conjunctival epithelial side population cells in humans, see GSE12631 (Akinci et al., 2009).

**Supplemental Text References**

Akinci, M. A., Turner, H., Taveras, M., Barash, A., Wang, Z., Reinach, P., & Wolosin, J. M. (2009). Molecular profiling of conjunctival epithelial side-population stem cells: atypical cell surface markers and sources of a slow-cycling phenotype. *Invest Ophthalmol Vis Sci, 50*(9), 4162-4172. doi:10.1167/iovs.08-2861

Barbato, S., Marrocco, E., Intartaglia, D., Pizzo, M., Asteriti, S., Naso, F., . . . Conte, I. (2017). MiR-211 is essential for adult cone photoreceptor maintenance and visual function. *Sci Rep, 7*(1), 17004. doi:10.1038/s41598-017-17331-z

Boneva, S., Schlecht, A., Bohringer, D., Mittelviefhaus, H., Reinhard, T., Agostini, H., . . . Lange, C. (2020). 3' MACE RNA-sequencing allows for transcriptome profiling in human tissue samples after long-term storage. *Lab Invest, 100*(10), 1345-1355. doi:10.1038/s41374-020-0446-z

Chen, D., Sahin, A., Kam, W. R., Liu, Y., Darabad, R. R., & Sullivan, D. A. (2018). Influence of lipopolysaccharide on proinflammatory gene expression in human corneal, conjunctival and meibomian gland epithelial cells. *Ocul Surf, 16*(3), 382-389. doi:10.1016/j.jtos.2018.05.003

Derrick, T., Habtamu, E., Tadesse, Z., Callahan, E. K., Worku, A., Gashaw, B., . . . Burton, M. J. (2019). The conjunctival transcriptome in Ethiopians after trichiasis surgery: associations with the development of eyelid contour abnormalities and the effect of oral doxycycline treatment. *Wellcome Open Research, 4*, 130. doi:10.12688/wellcomeopenres.15419.1

Diehn, J. J., Diehn, M., Marmor, M. F., & Brown, P. O. (2005). Differential gene expression in anatomical compartments of the human eye. *Genome Biol, 6*(9), R74. doi:10.1186/gb-2005-6-9-r74

Gipson, I. K. (2016). Goblet cells of the conjunctiva: A review of recent findings. *Prog Retin Eye Res, 54*, 49-63. doi:10.1016/j.preteyeres.2016.04.005

Gupta, D., Harvey, S. A., Kaminski, N., & Swamynathan, S. K. (2011). Mouse conjunctival forniceal gene expression during postnatal development and its regulation by Kruppel-like factor 4. *Invest Ophthalmol Vis Sci, 52*(8), 4951-4962. doi:10.1167/iovs.10-7068

Hori, Y., Spurr-Michaud, S. J., Russo, C. L., Argueso, P., & Gipson, I. K. (2005). Effect of retinoic acid on gene expression in human conjunctival epithelium: secretory phospholipase A2 mediates retinoic acid induction of MUC16. *Invest Ophthalmol Vis Sci, 46*(11), 4050-4061. doi:10.1167/iovs.05-0627

Hou, A., Lan, W., Law, K. P., Khoo, S. C., Tin, M. Q., Lim, Y. P., & Tong, L. (2014). Evaluation of global differential gene and protein expression in primary Pterygium: S100A8 and S100A9 as possible drivers of a signaling network. *PLoS One, 9*(5), e97402. doi:10.1371/journal.pone.0097402

Hu, V. H., Weiss, H. A., Ramadhani, A. M., Tolbert, S. B., Massae, P., Mabey, D. C., . . . Burton, M. J. (2012). Innate immune responses and modified extracellular matrix regulation characterize bacterial infection and cellular/connective tissue changes in scarring trachoma. *Infect Immun, 80*(1), 121-130. doi:10.1128/IAI.05965-11

Janssen, S. F., Gorgels, T. G., Bossers, K., Ten Brink, J. B., Essing, A. H., Nagtegaal, M., . . . Bergen, A. A. (2012). Gene expression and functional annotation of the human ciliary body epithelia. *PLoS One, 7*(9), e44973. doi:10.1371/journal.pone.0044973

Khandelwal, P., Liu, S., & Sullivan, D. A. (2012). Androgen regulation of gene expression in human meibomian gland and conjunctival epithelial cells. *Mol Vis, 18*, 1055-1067.

Latta, L., Ludwig, N., Krammes, L., Stachon, T., Fries, F. N., Mukwaya, A., . . . Kasmann-Kellner, B. (2021). Abnormal neovascular and proliferative conjunctival phenotype in limbal stem cell deficiency is associated with altered microRNA and gene expression modulated by PAX6 mutational status in congenital aniridia. *Ocul Surf, 19*, 115-127. doi:10.1016/j.jtos.2020.04.014

Majo, F., Rochat, A., Nicolas, M., Jaoude, G. A., & Barrandon, Y. (2008). Oligopotent stem cells are distributed throughout the mammalian ocular surface. *Nature, 456*(7219), 250-254. doi:10.1038/nature07406

Martin, G., Wolf, J., Lapp, T., Agostini, H. T., Schlunck, G., Auw-Hadrich, C., & Lange, C. A. K. (2021). Viral S protein histochemistry reveals few potential SARS-CoV-2 entry sites in human ocular tissues. *Sci Rep, 11*(1), 19140. doi:10.1038/s41598-021-98709-y

Master, A., Kontzias, A., Huang, L., Huang, W., Tsioulias, A., Zarabi, S., . . . Rigas, B. (2021). The transcriptome of rabbit conjunctiva in dry eye disease: Large-scale changes and similarity to the human dry eye. *PLoS One, 16*(7), e0254036. doi:10.1371/journal.pone.0254036

Nakatsu, M. N., Vartanyan, L., Vu, D. M., Ng, M. Y., Li, X., & Deng, S. X. (2013). Preferential biological processes in the human limbus by differential gene profiling. *PLoS One, 8*(4), e61833. doi:10.1371/journal.pone.0061833

Ramirez-Miranda, A., Nakatsu, M. N., Zarei-Ghanavati, S., Nguyen, C. V., & Deng, S. X. (2011). Keratin 13 is a more specific marker of conjunctival epithelium than keratin 19. *Mol Vis, 17*, 1652-1661.

Roberts, C., Franklin, C. S., Makalo, P., Joof, H., Sarr, I., Mahdi, O. S., . . . Holland, M. J. (2015). Conjunctival fibrosis and the innate barriers to Chlamydia trachomatis intracellular infection: a genome wide association study. *Sci Rep, 5*, 17447. doi:10.1038/srep17447

Tong, L., Diebold, Y., Calonge, M., Gao, J., Stern, M. E., & Beuerman, R. W. (2009). Comparison of gene expression profiles of conjunctival cell lines with primary cultured conjunctival epithelial cells and human conjunctival tissue. *Gene Expr, 14*(5), 265-278. doi:10.3727/105221609788681231

Turner, H. C., Budak, M. T., Akinci, M. A., & Wolosin, J. M. (2007). Comparative analysis of human conjunctival and corneal epithelial gene expression with oligonucleotide microarrays. *Invest Ophthalmol Vis Sci, 48*(5), 2050-2061. doi:10.1167/iovs.06-0998

Wong, Y. W., Chew, J., Yang, H., Tan, D. T., & Beuerman, R. (2006). Expression of insulin-like growth factor binding protein-3 in pterygium tissue. *Br J Ophthalmol, 90*(6), 769-772. doi:10.1136/bjo.2005.087486
